# Characterization of energy and performance of swine fed a novel corn-soybean extruded product

**DOI:** 10.1186/s40104-015-0011-6

**Published:** 2015-04-16

**Authors:** Katherine M Koch, Robert C Thaler, Sam K Baidoo, Crystal L Levesque, Rebecca C Bott

**Affiliations:** South Dakota State University, Brookings, SD 57007 USA; University of Minnesota Southern Research and Outreach Center, Waseca, MN 56093 USA

**Keywords:** Corn, Extruded, Lactation, ME, Soybean, Swine

## Abstract

**Background:**

A novel extruded product was characterized with a metabolism and lactation trial to establish the product’s energy content, and its effects on lactating sow performance. The product was composed of a 60:40 corn-soybean blend, which was then extruded. This product containing the co-extruded 60:40 corn-soybean blend was commercially developed and is used extensively in swine diets in southwest Minnesota. GE of dietary treatments were determined by isoperibol bomb calorimetry. Twelve barrows (59.9 ± 1.4 kg), were used to determine the digestible and metabolizable energy of the extruded product. DE of treatments was determined by subtracting fecal energy from GE provided to barrows by each respective treatment. ME was determined by subtracting urinary energy from calculated digestible energy. Sixty-three sows were used for the lactation trial. Three dietary treatments were utilized: CONTROL (an industry standard diet); PRODUCT (contained the product, vitamins and minerals); OIL (matched the lysine:ME ratio of PRODUCT by addition of soy oil). Sow weight, backfat thickness at the right and left last ribs, body condition score, number of piglets, and litter weights were recorded on the date of farrowing (d 0), (d 9), and at weaning. Blood and milk samples were obtained at weaning, and blood was analyzed for plasma urea nitrogen (PUN), milk was analyzed for total protein and fat content.

**Results:**

On a dry-matter basis, the test diet provided 3,908 kcal/kg DE and 3,833 kcal/kg ME, which was significantly greater than the basal diet, which provided 3,633 kcal/kg DE and 3,567 kcal/kg ME (*P* < 0.0001). These data were used to establish the DE and ME of the product, which were 3,882 kcal/kg and 3,798 kcal/kg, respectively, on an as-fed basis. No effect of diet was observed for changes in sow backfat (RBF *P* = 0.24; LBF *P* = 0.07) or body condition score (*P* = 0.12) during lactation. Milk total protein (*P* = 0.69), fat (*P* = 0.66), PUN, average piglet gain (*P* = 0.55) and piglet mortality (*P* = 0.70) did not differ between treatments.

**Conclusions:**

While the novel extruded product was higher in energy content than traditional feedstuffs, it resulted in the same lactational sow performance. Thus, the co-extruded corn-soybean product is a reasonable inclusion in sow lactation diets.

## Background

It is well established that feed accounts for the largest portion of production costs for a livestock enterprise, and energy is an expensive component of feed [1]. Therefore, any improvement in feed efficiency should decrease feed costs per animal. Corn and soybeans are the base ingredients of U.S. swine rations [2] representing the primary energy and protein sources, respectively. Soybean meal (SBM) is heat-treated to inactivate the anti-nutritional factors in raw soybeans, and the majority of oil removed for commercial sale. Extrusion has also been shown to be an effective method to destroy anti-nutritional factors such as trypsin inhibitor in raw soybeans [3,4]. Additionally, extrusion improves energy digestibility in both soybeans [5] and corn [6,7] by initiation of starch gelatinization. Therefore, extruded soybeans may be effectively integrated into swine diets without sacrificing nutritional integrity due to inactivation of trypsin inhibitor and improved energy digestibility.

Extruding corn and soybeans together is expected to yield a product with higher energy digestibility than corn or SBM alone. Replacing corn and SBM in lactation diets with a corn-soybean extruded product may provide a way to improve sow energy intake during lactation and enhance piglet performance. Feed intake, or more specifically dietary energy intake, is closely related to both sow and litter performance [8]. A decrease in energy intake may inhibit litter growth potential and increase body weight loss of sows [9], whereas, litter gains were increased with increased dietary energy utilization in the sow [8]. Therefore, since it should be higher in energy content the extruded product’s use in lactation diets has been hypothesized to improve litter gains. It is hypothesized that replacing corn and SBM with a corn-soybean extruded product in sow lactation diet may improve sow and litter performance.

## Methods

Experiments were conducted in accordance with the South Dakota State University Institutional Animal Care and Use Committee (Protocol #10-080E).

### Exp. 1: determination of digestible and metabolizable energy of a corn-soybean extruded product

#### Animals

A total of 12 crossbred barrows (Yorkshire × Landrace) with an initial body weight of 59.9 ± 1.4 kg were used in a two period crossover design. Barrows were randomly blocked as pairs by crate assignments and randomly assigned to one of the two experimental diets as treatments within blocks. Period one of the experiment was a nine-day diet acclimation period followed by a four-day total collection of urine and feces. There was a two day rest between periods, with a dietary treatment switchback occurring at the start of period two, which also was comprised of a nine-day diet acclimation followed by a four-day total collection of urine and feces. At the start of each period, barrows were weighed and placed in individual, raised metabolism crates. Crate assignments and placement within the room were identical for periods one and two. Crates allowed for separate but complete fecal and urine collection.

#### Diets

A 60:40 corn-soybean blend was extruded to produce the extruded corn-soybean product used in the study. Proximate and lysine analysis of the corn-soybean extruded product is shown in Table 1. Composition and chemical analysis of diets are shown in Table 2. The basal diet was prepared at the SDSU Feed Mill and consisted of 97.1% corn and 2.9% vitamin and mineral premix that met or exceeded NRC [10] vitamin and mineral requirements for growing pigs. The test ingredient diet, (CSOY) was comprised of 70% basal diet with 30% of the corn soybean extruded test product, and was prepared using a Marion Mixer (Model number 2030, Rapids Machinery Co., Marion, IA, USA). Barrows were limit-fed to three percent of body weight and feed was provided in two equal daily allotments at 0800 h and 1600 h. Body weight used to calculate feeding level was measured at the start of each experimental period. Orts were collected and weighed before the morning feeding of the first day of each collection period and upon the completion of the experiment.

**Table 1:** **Composition analysis of corn-soybean extruded product for exp. 1 and 2 (AF basis)**

**Item**	**Exp. 1**	**Exp. 2**
CP, %	19.49	19.78
Crude fat, %	8.88	8.45
Crude fiber, %	2.63	2.81
Ash, %	2.81	3.06
Total lysine, %	1.02	1.09

Proximate analysis of diets was performed by SGS North America (Brookings, SD). Diet lysine analysis was performed by the University of Missouri Experiment Station Chemical Laboratories.

**Table 2:** **Composition of basal and test diets (AF basis)**

**Item**	**BASAL**	**CSOY**
Ingredient, %		
Corn	97.05	67.97
Extruded corn/soybeans	--	30.00
Dicalcium phosphate	1.22	0.85
Limestone	0.73	0.511
Sodium chloride	0.40	0.28
Vitamin and mineral premix^1^	0.60	0.42
Composition analysis^2^		
CP, %	8.29	11.33
Crude Fat, %	3.23	4.09
Crude Fiber, %	1.45	1.72
Ash, %	3.08	2.91
Total lysine, %	0.24	0.58

^1^Provided the following per kilogram of basal diet: vitamin A, 7,716 IU; vitamin D3, 1,929 IU; vitamin E, 39 IU; vitamin B_12_, 0.04 mg; riboflavin, 12 mg; niacin, 58 mg; pantothenic acid, 31 mg, copper, 35 mg; iron, 350 mg; iodine, 4 mg; manganese 120 mg; zinc, 300 mg, selenium, 0.3 mg.

^2^Proximate analysis of diets was performed by SGS North America (Brookings, SD). Diet lysine analysis was performed by the University of Missouri Experiment Station Chemical Laboratories.

### Fecal and urine collections

Total 24 h collection of feces and urine was performed immediately following morning diet allocations on each of the 4 collection days. Thirty mL of 6 N HCL was added to every urine collection pail at the start of each collection day after the previous day’s collection was complete. Urine was weighed and volumes recorded; 100 mL subsamples were frozen and stored at −20°C until later analysis. Collection of feces was initiated immediately following the morning feed allotment and ended 96 h later. Collected feces were weighed and stored at −20°C. At the end of each experimental period, total collected feces were pooled within barrow and remained frozen until later analysis. Fecal dry matter was determined by first, thawing and mixing pooled feces within barrow then, drying a 1 kg subsample in a forced air oven (Model number LHD 2–14, Despatch Oven Co., Minneapolis, MN, USA) at 65°C for 48 h.

### Energy analysis

Isoperibol bomb calorimetry (Parr 1261 Isoperibol Bomb Calorimeter, Parr Instrument Co., Moline, IL, USA) was used to determine gross energy (**GE**) of the novel product, corn, soybeans, feces and urine, with benzoic acid used as a standard. Feedstuffs and feces were ground through a 1 mm screen. Feed and feces were processed according to standard protocol (AOAC, 1990) prior to energy determination. Prior to energy calculation, 10 mL of urine was pipetted into plastic bags containing cotton, frozen at −80°C for 24 h and subjected to freeze drying conditions for 36 h. Both plastic bags and cotton weights were measured for each sample. Cotton and bag GE was determined by isoperibol bomb calorimetry, These calculated values were then used to extrapolate actual urine GE from sample energy analysis. All samples were analyzed in duplicate.

### Calculations

Individual barrows were considered the experimental units. GE intake for each barrow was calculated by multiplying total dry matter (DM) intake over the four day collection period by feedstuff GE content determined from isoperibol bomb calorimetry analysis. Diet digestible energy (DE) was calculated by subtracting fecal energy from GE. Diet metabolizable energy (ME) was calculated by subtracting urinary energy from DE. Apparent DE and ME of the corn-soybean product were extrapolated by difference from that of the basal diet, with basal diet DE and ME subtracted from the DE and ME of the CSOY diet [11,12].

Data were analyzed using PROC MIXED of SAS (SAS Inst. Inc. Cary, NC, USA). Barrow was considered the experimental unit. Observations from every barrow were included in data analysis, as none were removed from the trial. The model included the main effects of dietary treatment, experimental period, and dietary treatment x experimental period interaction. Block was considered random. Least squares means were considered significant when *P* ≤ 0.05.

### Exp. 2: evaluation of an extruded corn-soybean product in sow lactation diets

#### Animals

A total of 51 mixed parity (0–9) sows (Yorkshire × Landrace; Topigs 20, Winnipeg, MB, Canada) and their respective litters were used in this lactation study. The sow herd became infected with PRRS in November 2011, six months before the experiment started but had stabilized by the initiation of the trial. Sows were bred with pooled Duroc semen (Compart’s Boar Store, Inc., Nicollet, MN, USA) and originated from a single lactation group of 63 sows. The study was performed in a completely randomized design with sow being the experimental unit. Sows were randomly assigned to one of 3 dietary treatments, and provided 1.4 kg rations each day for 5 d prior to expected farrowing date as an adjustment period before the start of the experiment. Upon the conclusion of the 5 d adjustment period, 12 sows were removed from the experiment due to feed refusal or health complications. The trial lasted the length of lactation, which was standardized to 18 d (lactation length ranged from 17–23 d). Piglets were cross fostered within dietary treatment groups within three days post-farrowing. Piglets/litter was the same for the sows in each respective treatment but not necessarily equal across all dietary treatments as we did not have the same number of pigs farrowed for each dietary treatment since we culled sows. The average number of piglets/litter was 12.1, 11.6 and 11.7 for treatments 1, 2, and 3, respectively.

#### Dietary treatments

A new batch of corn-soybean extruded product was produced for the lactation performance study and proximate and lysine analysis of the corn-soybean extruded product used in Exp. 2 are shown in Table 1. The control diet, (CONTROL, n = 18 sows), was formulated to represent a standard industry corn-soybean meal based lactation diet that met or exceeded NRC [10] nutrient recommendations for lactating sows (Table 3). The second diet (PRODUCT, n =15 sows) was comprised of the corn-soybean extruded product with the addition of vitamins and minerals to meet or exceeded NRC [10] recommendations (Table 3). This is a new diet being used in southwestern Minnesota. The third diet (OIL, n = 18 sows), was formulated to be similar to that of CONTROL, with the addition of soybean oil to equal the lysine:Mcal ME ratio of PRODUCT (Table 3). The reason the third diet was added was to be able to separate out a fat versus extrusion effect. Since the PRODUCT and OIL diets contained the same lysine:calorie ratio, if there was no difference between them, then the effect was due to added fat. However, if there was a difference between the PRODUCT and OIL diets, then the effect could be attributed to extrusion. Due to bin space constraints, the diets were prepared in two batches, with the first batch prepared eight days prior to the beginning of the experiment and the second batch prepared and dispensed six days prior to weaning. Diet composition analyses of both batches are shown in Table 4. Sows were provided diet allotments in morning and afternoon feedings by hand in a step-up manner to achieve ad-libitum feed intake. Sows were split-rationed 1.4 kg daily rations at the start of the experiment; feed additions were increased in 0.45 kg increments at each feeding if no orts were collected. Feed additions were weighed and recorded daily.

**Table 3:** **Composition of lactation diets (AF basis)**

**Item**	**Diet**		
	**Control**	**Product**	**Oil**
Ingredient, %			
Corn	67.28	--	57.69
Soybean meal, 47%	29.15	--	30.95
Extruded corn-soybeans	--	96.45	--
Soy oil	--	--	7.8
Dicalcium phosphate	1.50	1.40	1.50
Limestone	1.22	1.30	1.21
Salt	0.50	0.50	0.50
Vitamin and trace mineral premix^1^	0.35	0.35	0.35
Calculated analysis			
CP, %	19.10	18.50	19.20
Crude fat, %	3.50	11.00	11.00
Ca, %	0.84	0.84	0.84
Total P, %	0.70	0.65	0.69
Available P, %	0.39	0.39	0.39
Total lysine, %	1.04	1.02	1.07
SID lysine, %	0.90	0.93	0.93
SID lysine: Mcal ME	2.76	2.54	2.54

^1^The vitamin and trace mineral premix provided the following (per kg of diet): vitamin A, 11,000 IU; vitamin D_3_, 2,756 IU; vitamin E, 55 IU; vitamin B_12_, 55 μg; vitamin K, 4.4 mg riboflavin, 9.9 mg; pantothenic acid, 33 mg; niacin, 55 mg; choline, 495 mg; pyridoxine, 2.2 mg; folic acid, 1.65 mg; thiamine, 1.1 mg; biotin, 220 μg; I, 2.2 mg; Zn, 150 mg; Fe, 124 μg; Mn, 40 mg; Cu, 14.9 mg; and Se, 0.3 mg.

**Table 4:** **Composition analysis of lactation diets (AF basis)** ^**1**^

**Item**	**Batch 1** ^**a**^			**Batch 2** ^**a**^		
	**Control**	**Product**	**Oil**	**Control**	**Product**	**Oil**
Proximate analysis^2^						
CP, %	17.05	19.58	18.93	15.72	18.85	18.28
Crude fat, %	2.60	8.58	9.34	2.85	8.37	9.14
Crude fiber, %	2.25	3.00	2.84	1.99	2.39	1.92
Ash, %	3.70	3.61	4.85	3.74	4.46	4.08
Lysine^3^						
Total lysine, %	0.94	1.01	0.95	0.95	1.05	1.00

^1^Diets were mixed and prepared at U of Minnesota SROC.

^2^Proximate analysis of diets was performed by SGS North America (Brookings, SD).

^3^Lysine analysis was performed by the University of Missouri Laboratory.

^a^Batch 1 was prepared 8 d before the experiment started. Batch 2 was prepared and dispensed 6 d before weaning.

#### Performance measurements

Weaning date for the experiment was predetermined before the start of the trial, according to the research facility swine production schedule. Lactation lengths ranged from 17–23 d and averaged 18.6, 18.3 and 18.6 d for CONTROL, PRODUCT and OIL, respectively. Sow body weight (BW, n = 51), body condition score (BCS, n = 51), and last rib backfat [6 cm to the left (LRBF) and 6 cm to the right (RRBF) of the midline] were taken at days 0, 9 and at weaning. Day 0 was designated as day of farrowing. Backfat measurements were taken with a Renco Lean o’ Meter backfat ultrasound (Renco Corporation, Minneapolis, MN). Body condition scoring was performed by a single trained technician in 0.5 increments on a scale of 1 to 5 in the manner described by Coffey et al. [13] and Patience and Thacker [14], where a score of 1 represents a very thin sow, and a score of 5 represents an obese sow. Sow feed intake and orts were recorded daily.

The number of piglets born alive per litter was recorded and litter weight was measured at days 0, 9 and at weaning. Piglets were weighed collectively in their litters in a portable box scale. Cross fostering of piglets occurred within treatment and within three days of farrowing; cross fostered piglets were individually weighed, and weights were deducted from original litters and added to their respective new ones. Litter gain (LG) was calculated as the change in litter weight from day 0, 9 and weaning, adjusted for the number of piglets per litter at each point.

### Milk and blood collection

At weaning, eight sows were randomly selected from each dietary treatment for blood collection. However, not all sows were utilized due to facility limitations and a total of twenty sows were used for blood and milk sampling (CONTROL, n = 6; PRODUCT, n = 6; OIL, n = 8). Immediately following piglet removal at weaning, selected sows were snared and 10 mL of blood was collected via jugular venipuncture in non-heparinized collection tubes (BD Vaccutainer, Franklin Lakes, N.J., U.S.A.). Sows were then given 20 USP of oxytocin via intramuscular injection directly in the udder to initiate milk letdown. Milk was hand stripped from all functional teats until approximately 80 mL was collected. Blood was centrifuged and the plasma portion collected and frozen at −20°C until analysis for plasma urea nitrogen (PUN). Milk subsample aliquots (10 mL) were frozen and stored at −20°C until analysis for fat and total protein concentration.

All data were analyzed using the PROC MIXED procedure of SAS (SAS Inst. Inc. Cary, NC, USA). Sow was considered the experimental unit. The model statement for analyses of overall sow and litter criteria for the lactation period, as well as milk composition and PUN concentration, included the main effects of diet, replication, and parity as a covariate. Time, representative of day of data collection, was also included in the model statement for separate analyses of sow performance criteria within the lactation period. Orthogonal contrasts were used to compare differences from diets of lower (CONTROL) energy density to that of elevated energy density (PRODUCT, OIL), as well as differences between the two diets of elevated energy content (PRODUCT, OIL).

## Results and discussion

### Exp. 1

The energy of diets and the extruded corn-soybean product are reported in Table 5. Extrusion processing improved energy digestability, as is evident by increased GE, DE, and ME for the test diet, compared to the basal diet (Table 5; *P* = 0.001). Friesen et al. [15] directly compared diets containing extruded soybean products to non-extruded corn and soybean based diets and reported improved ADFI, ADG and gain:feed ratios by piglets provided diets containing extruded soy products.

**Table 5:** **Energy content of diets and extruded corn-soybean product (AF basis)**

**Item**	**BASAL diet**	**CSOY diet**	***SEM***	**Product**
**Energy**				
GE, kcal/kg^1^	3,791	3,979	--	4,514
DE, kcal/kg^,2,3^	3,239	3,473	25	4,114
ME, kcal/kg^,2,4^	3,177	3,409	27	4,045
ME, % of GE^5^	84	86	0.61	90

^1^GE was calculated by isoperibol bomb calorimetry.

^2^Product DE and ME were calculated by difference from the basal diet.

^3^ *P*-values: Diet = 0.0001; period = 0.1786; period*diet = 0.7706.

^4^ *P*-values: Diet = 0.0001; period = 0.2265; period*diet = 0.5595.

^5^ *P*-values: Diet = 0.0577; period = 0.1779; period*diet = 0.5805.

Extrusion processing also tended (*P* = 0.06) to improve the ME as a percent of DE in the test diet (86%) and product (90%), compared to the basal diet (84%). The ME:DE ratio of the basal diet was 98%, which is similar to the 97% ME:DE for corn [10]. As the basal diet was almost completely comprised of corn (97.1%), its ME:DE ratio was expected to closely reflect that of corn, therefore, the experiment’s basal diet ME and DE calculations supported this hypothesis.

The DE and ME values for corn, on a dry matter basis, provided in the NRC [10] are 3,961 and 3,843 kcal/kg. On a DM basis, the basal diet contained 3,639 kcal/kg DE and 3,570 kcal/kg ME. Adjustment of the NRC [10] corn DE and ME values for the 97.1% of the basal diet composed of corn, results in calculated basal diet estimations of 3,846 kcal/kg DE and 3,731 kcal/kg ME, which is slightly higher than observed in the current study. However, since the ME:DE ratio was similar for the experiment’s calculations to that reported by the NRC [10], it may be suggested that the corn used in the experiment may have been slightly poorer in quality and contained slightly less energy than expected. It must also be noted that the basal diet, comprised almost entirely of corn, was not properly balanced for amino acids, especially lysine. It may be possible that calculated ME values for the experiment may be slightly underestimated, due to possible increased urinary nitrogen excretion compared to balanced diets [1].

Calculated DE and ME values for the extruded corn-soybean product are in agreement with the DE and ME value ranges reported by Anderson et al. [1], who measured the energy content of several corn products and Woodworth et al. [16], who measured energy content of extruded soybean meal. DE and ME values of meal from extruded soybeans without hulls were calculated to be 4,210 kcal/kg DE and 3,960 kcal/kg ME on an as-fed basis [16]. Anderson et al. [1] calculated low fiber corn products (corn starch, dried corn solubles, de-hulled, de-germed corn and corn oil) to contain between 4,082 to 8,988 kcal/kg DE and 4,080 to 8,755 kcal/kg ME on a dry-matter basis. For comparison, the corn-soybean extruded product used in the current study contained 4,622 kcal/kg DE and 4,545 kcal/kg ME on a dry-matter basis and 4,114 kcal/kg DE and 4,045 kcal/kg ME on an as-fed basis. The swine NRC [10] recommends DE and ME content of growing pig diets to contain at least 3,400 kcal/kg and 3,265 kcal/kg (AF) for ad libitum feeding; therefore, the extruded product may be fed as an appropriate energy-dense feedstuff for growing-finishing swine.

### Exp. 2

It must be noted that sows in the production facility in which the experiment was conducted suffered an outbreak of PRRS six months prior to the start of the experiment, in November of 2011. Therefore, it may be possible that any improvements in performance, attributed the extruded product, may have been hindered by the reduced herd health status of the production facility.

The effects of extruded corn-soybean product inclusion in sow lactation diets on sow and litter criteria are reported in Table 6. No effect due to dietary treatment was observed in backfat (RBF *P* = 0.24; LBF *P* = 0.07) or body condition score (*P* = 0.12) changes over the total lactation period.

**Table 6:** **Effect of diet on sow and litter criteria** ^**1**^

**Item**	**Diet**				***P*** **-values** ^**3**^	
	**Control**	**Product**	**Oil**	**SEM** ^**2**^	**I**	**II**
No. Sows	18	15	18	--	--	--
ADFI, kg	4.52	5.77	4.98	0.40	0.05	0.51
Sow weight, kg						
D 0	259.02	258.81	262.11	6.82	0.31	0.53
D 9	257.49	259.47	256.14	5.72	0.67	0.22
Weaning	249.23	258.51	248.20	6.28	0.96	0.15
Right backfat, mm						
D 0	21.70	19.48	20.81	1.19	0.75	0.83
D 9	20.28	18.74	19.46	1.00	0.55	0.37
Weaning	19.32	17.81	18.90	1.00	0.63	0.63
Overall change	−2.38	−1.66	−1.92	0.64	0.88	0.72
Left backfat, mm						
D 0	21.87	19.42	20.55	1.20	0.58	0.80
D 9	20.50	19.01	18.92	1.14	0.40	0.24
Weaning	19.42	18.25	18.91	0.98	0.76	0.42
Overall change	−2.23	−1.29	−1.71	0.64	0.58	0.28
Sow BCS^4^						
D 0	3.01	2.80	3.03	0.11	0.23	0.99
D 9	2.90	2.82	2.92	0.09	0.53	0.71
Weaning	2.98	2.82	2.89	0.09	0.26	0.70
Overall change	0.01	0.00	−0.15	0.12	0.81	0.93
APG^5^, kg	4.33	5.48	4.81	0.22	0.48	0.33
Litter weight, kg						
D 0	18.00	17.14	19.23	0.59	0.07	0.23
D 9	39.06	37.78	41.46	1.58	0.74	0.41
Weaning	68.52	72.04	72.33	2.89	0.89	0.73
Litter mortality, %	1.97	11.03	4.63	2.08	0.94	0.41

^1^Data are presented are LS means*.*

^2^Standard error reported is largest of diet LS means.

^3^ *P*-values reported are of contrast I (Control vs Product + Oil) and contrast II (Product vs Oil).

^4^Body condition score = determined on a 5 point scale with 0.5 point increments by a single person (Coffey, [13]; Patience, [14]), with a score of 1 representing poor condition, 5 representing over-condition and 3 considered ideal.

^5^Average piglet gain = litter gain averaged for treatment replications and adjusted for number of live piglets at each measurement.

Sows provided PRODUCT exhibited a similar weight to CONTROL sows or those fed OIL at D0, D9, and at weaning. Parity was balanced similarly across dietary treatments to the best extent possible, as the sow herd was of mixed parities with an uneven parity distribution. Additionally, average piglet gains, litter weights at D0, 9 and weaning and percent mortality losses per litter did not differ between dietary treatments.

Sow average daily feed intake (ADFI) was lower for CONTROL (4.52 kg), when compared simultaneously with PRODUCT (5.77 kg) and OIL (4.98 kg; *P* = 0.05) and overall sow weight change was greater for CONTROL (−10.35 kg) compared to PRODUCT (+0.02 kg) and OIL (−13.71 kg; *P* = 0.05). This may have resulted from the depressed feed intake exhibited by sows fed CONTROL, as sows would have elevated body store mobilization to compensate for reduced energy intake in order to meet lactation demands, as described by Kim and Easter [17]. No differences due to dietary treatment were detected in Contrast II (PRODUCT vs OIL) for overall sow weight change. It may be hypothesized that the lack of difference may have resulted from sows’ consumption of similar amounts of calories as ADFI did not differ and lysine:ME was constant between PRODUCT and OIL, therefore sows may have mobilized similar amounts of body stores to support lactation demands. Additionally, the sow herd used in the experiment was PRRS+, which may account for the overall low feed intakes exhibited by the sows.

The effects of extruded corn-soybean product inclusion in sow lactation diets on sow milk composition are reported in Table 7. There was no difference in milk total protein, percent milk fat, or sow plasma urea nitrogen (PUN) between dietary treatments. However, PUN was altered by elevated dietary energy, as it was increased when PRODUCT and OIL were compared to CONTROL (*P* = 0.02).

**Table 7:** **Effect of diet on sow milk composition and plasma urea nitrogen** ^**1**^

**Item**	**Diet**				***P*** **-values** ^**3**^	
	**Control**	**Product**	**Oil**	**SEM** ^***2***^	**I**	**II**
No. Sows	6	6	8	--	--	--
Total protein^4^, %	5.27	5.20	5.09	0.16	0.50	0.60
Fat, %	7.8	7.36	8.0	0.59	0.85	0.39
PUN^5^, mg/dL	15.49	19.56	20.8	1.78	0.02	0.59

^1^Data presented are LS means.

^2^Standard error reported is largest of diet LS means.

^3^ *P*-values reported are of contrast I (Control vs Product + Oil) and contrast II (Product vs Oil).

^4^Expressed as N × 6.38.

^5^Plasma urea nitrogen.

Extrusion did not improve the energy utilization of corn and soybean used in a lactation diet, compared to a diet matched with soybean oil on a lysine:ME ratio. Sow productivity is directly related to both ME and lysine; which levels influence the utilization of each other [18]. This experiment balanced lysine:ME ratios for the energy-dense diets to see if the extrusion processing of PRODUCT would improve performance above that of OIL, when this ratio was held constant (Table 3).

Sows demonstrated increased ADFI (Table 6) for the energy-dense dietary treatments. Sows used in the experiment were of average body condition across all dietary treatments (Table 6) for the experiment, according to the scale described by Coffey et al. [13] and Patience and Thacker [14]; sows of normal body condition increase milk production when feed consumption is increased [19].

Milk fat from sows fed CONTROL were similar to values from non-fat supplemented sows previously reported [3,20,21]. However, the lack of difference in milk fat content of the experimental diets used in the current study is in contrast to Seerley et al. [22] and Shurson et al. [20], who demonstrated that increased dietary energy in the form of fat increases milk fat content. However, the similarity of milk fat in the PRODUCT and OIL dietary treatments (Contrast II, *P* = 0.39) agrees with the work of Revell et al. [23], who established that milk composition is not altered by diets of similar energy content.

Total protein values in milk were similar to values reported by previous research [24]. The similarity of total milk protein content for dietary treatments (Table 7; Diet *P* = 0.69) was expected, based on the data presented by Seerley et al. [22], who demonstrated no change in milk protein as a result of added dietary fat. Additionally, Klaver et al. [19] established that milk compositional changes are attributed to body condition changes, and are not reflective of diet changes. It must also be noted that the dietary treatments were not provided during gestation; therefore body reserves of sows utilized in the experiment would have been reflective of the gestational diet and not experimental dietary treatments. Revell et al. [23] clearly states that milk substrates may be derived from either feed or body reserves, therefore a lack of milk compositional changes may have resulted from the sows’ uniformity of body reserves. It is also possible that the dietary treatments used in the experiment did not differ enough nutritionally to have presented effects in milk composition.

PUN analysis has been widely accepted within the swine industry as a rapid response criterion for determination of feedstuff amino acid utilization, specifically that of lysine [25-28]. PUN concentrations are reflective of protein metabolism [26].

PUN data did not differ between PRODUCT and OIL treatments (Table 7; Contrast II *P = *0.59). Nitrogen retention, determined by PUN analysis, was influenced by dietary fat source [29]. This may explain the elevation of PUN for PRODUCT and OIL, compared to that of CONTROL (Contrast I *P = *0.02), and concurrently, the similarity between the energy-dense diets (Contrast II *P = *0.59), as they both contained soy-derived fat (Table 7).

PUN data yielded from the present experiment is likely a reflection of the higher feed intake. (Table 7; Contrast I *P =* 0.02). PUN concentrations from the present experiment were higher than those reported by Coma et al. [26]. However, Coma et al. [26] stated that lysine provided above nutrition requirements will increase PUN concentrations. The failure of energy-dense, PRODUCT and OIL, to improve litter weight at weaning over that of CONTROL (Contrast I, *P =* 0.89) is not in agreement with previous research that reported increased litter gains when sows were fed energy-dense lactation diets [30,31]. However, this may be attributed to the sows’ ADFI, as low feed intakes inhibit litter gain [9]; sows in the experiment did not consume enough feed to obtain lysine intakes required to maximize litter growth, based on the estimations of Coma et al. [26]. Coma et al. [27] reported that modern, adult sows supporting litters of 10 piglets require 55 g/d of lysine to support 2.2 kg of litter growth per day. The sows in this experiment approximately consumed an average of 5.09 kg of feed each day, with an average of 0.96% lysine availability. Therefore, they consumed approximately 49 g of lysine/day. Therefore, it is possible that sows’ ADFI, and therefore lysine intake, could have limited piglet growth in the present experiment. However, it must be noted that PRODUCT did not result in depressed litter gains, as no differences in litter weights at weaning were detected at *P* ≤ 0.05. Therefore, the extruded product yielded similar litter gains to that of standard lactation diet.

PUN data yielded from the present experiment is likely a reflection of the higher feed intake. (Table 7; Contrast I *P =* 0.02). PUN concentrations from the present experiment were higher than those reported by Coma et al. [26]. However, Coma et al. [26] stated that lysine provided above nutrition requirements will increase PUN concentrations. The failure of energy-dense, PRODUCT and OIL, to improve litter weight at weaning over that of CONTROL (Contrast I, *P =* 0.89) is not in agreement with previous research that reported increased litter gains when sows were fed energy-dense lactation diets [30]. However, this may be attributed to the sows’ ADFI, as low feed intakes inhibit litter gain [9]; sows in the experiment did not consume enough feed to obtain lysine intakes required to maximize litter growth, based on the estimations of Coma et al. [26]. Coma et al. [27] reported that modern, adult sows supporting litters of 10 piglets require 55 g/d of lysine to support 2.2 kg of litter growth per day. The sows in this experiment approximately consumed an average of 5.09 kg of feed each day, with an average of 0.96% lysine availability. Therefore, they consumed approximately 49 g of lysine/day. Therefore, it is possible that sows’ ADFI, and therefore lysine intake, could have limited piglet growth in the present experiment. However, it must be noted that PRODUCT did not result in depressed litter gains, as no differences in litter weights at weaning were detected at *P* ≤ 0.05. Therefore, the extruded product yielded similar litter gains to that of standard lactation diet.

## Conclusions

Inclusion of the extruded corn-soybean product in lactation diets resulted in similar sow and litter performance to that of a standard corn-soybean meal lactation diet. However, the extruded corn-soybean product did not improve performance over that of an equal lysine:energy corn-soybean meal diet supplemented with soy oil. The current data supports the product’s suitability as a feedstuff for swine lactation diets,. Additional experiments to fully determine the impacts of extruding corn and soybeans together to potentially improve swine performance are warranted.

## Abbreviations

GE: Gross energy
DE: Digestible energy
Kcal: Kilocalorie
Kg: Kilogram
ME: Metabolizable energy
D: Day
PUN: Plasma urea nitrogen
RBF: Right rib back fat
LBF: Left rib back fat
SBM: Soybean meal
SDSU: South Dakota State University
NRC: National research council
CSOY: Test ingredient
N: Normal
HCL: Hydrochloric acid
DM: Dry matter
BCS: Body condition score
Cm: Centimeter
BW: Body weight
ADFI: Average daily feed intake
ADG: Average daily gain
AF: As fed
